# A Description of the Summer and Autumnal Diseases as They Occur in the Wabash Valley, and Particularly in Posey County, Indiana, and in White County, Illinois

**Published:** 1843-09

**Authors:** Robert Robson

**Affiliations:** New Harmony, Indiana


### Art. II.—*A description of the Summer and Autumnal Diseases as they occur in the Wabash Valley, and particularly in Posey County, Indiana, and in White County, Illinois.* By Robert Robson, M.D., of New Harmony, Indiana.

The valley of the Wabash furnishes the most extensive field for observation on all the grades of our summer and autumnal diseases, with which I am acquainted, and it may not be altogether uninteresting to the profession for me to state what my experience has taught me on that subject, during a residence of eighteen years in New Harmony, Posey county, Indiana.

*Topography.*—The Wabash is a beautiful river, running generally south. Its banks are low and extend back in a rich, level plain from three to five miles. Harmony is situated back from the river about a quarter or half a mile, on a second bank, and about six or eight feet above high water mark. On the opposite side the ground for many miles above and below this point is four or five miles wide. The lands adjacent to the river are higher than those in the rear. Numerous small bayous lead back and fill up the ponds and cover the low grounds. The face of the country for many miles above and below, in the neighborhood of the river, is generally level. The soil is alluvial and very rich, consisting of sand and black loam. The bottoms are covered with a heavy growth of timber, such as is peculiar to a rich soil, and the great mass of the lands lying opposite the village remains very generally in the natural state. Such of the above lands as are not in cultivation, are, after the month of May, covered with a very rank growth of vegetation of every kind. There are generally high waters at some period during the spring months, or between the first of March and the month of June; this has never failed since my residence here. Some years there are two freshets. In 1834 and 1836 the second came in the last of May or first of June.

When I commenced practice here in 1830 or 1831, I found the country, with the exception of the town of *New Harmony, which has been for many years remarkable for the health of its citizens,* infested with fevers almost of every grade from the most simple intermittent to the most fatal remittent. The spring opened with intermittents of a mild form accompanied occasionally with remittents. As the season advanced and the weather became warm, the severity and obstinacy of the intermittents became proportionably increased, and remittents became more severe and numerous. Towards autumn and during the fall, bilious remittents were very prevalent and often attended with symptoms of very great severity, developing in their course all the characteristics of western bilious fever. During its progress, if the patient had been neglected, or the treatment not well directed, the tongue became covered with a dry brown or black scurf, the skin dry and husky, with much disturbance of the sensorium sometimes, though not frequently. Some cases were of a lingering character, continuing for ten or twelve days, and then terminating favorably. The general character of the diseases, when suitable attention was paid, was to intermit from the fifth to the ninth day, and form a salutary crisis.

The constitutional type was the same during the three succeeding years, though there was less sickness in the last then in the two preceding, and that which occurred was of a much milder type. During the summer and autumn of 1834 very few families in this district of country escaped the visitation of fever, which generally assumed the most serious character, carrying off many of the most respected citizens. The weather of this year differed much from the preceding with respect to rain, dryness, heat, wind, &c., &c. The early part of spring was remarkably cool and pleasant, there were frequent rains of short continuance, and the country was more healthy than usual.

May—Very warm and dry—several cases of mild intermittent fever.

June—Ushered in with very warm and dry weather, vegetation abundant, the Wabash high, and in low places overflowing the banks, and inundating the low lands—after the 17th we had frequent and heavy rains—a few cases of cholera morbus occurred.

July—The sickly season, as it is familiarly called here, begins about this time and continues until the frosts in the fall. In this month there were very warm winds, generally from the south-west. Remote from the Wabash, on the adjacent high grounds, dysenteries and cholera morbus prevailed, but towards the latter part of July subsided; whilst in August intermittent and remittent fevers became general, and of a more severe grade.

September—The east-winds generally prevailed, with the usual temperature of August, until the latter part of the month, when it became much cooler, and the winds more north—a high grade of inflammatory fever prevailed. The congestive form became more general as the weather grew colder.

October—Winds generally west; heat of the sun oppressive during the middle of the day, with cool nights, until after the middle of the month, when the temperature seldom exceeded 50° Fah. After this time fevers rapidly subsided and were superceeded, in my practice, by a disease commonly denominated milk-sickness or trembles, which was at that time generally confined to neighborhoods bordering on the river.

In the above very brief, but I believe generally correct statement of the weather of the summer and fall of 1834, it will be observed why diseases are more violent in some seasons, and in some situations than in others—that the winds were mostly from a southern direction, while the low-lands were overflowed in the month of June when vegetation had much advanced, and in July were left with a large amount of putrefactive matter on the surface, to be acted upon by the heat of the sun, and consequently propagating miasmata and disease. In such seasons, we not only invariably have more sickness, but there is an increased malignancy in the character of the diseases.

The fevers of our summer and autumnal months are those generally denominated simple, inflammatory and congestive. The simple form of fever comes on with listlessness and languor, which sometimes continued two or three days, when the patient was taken with a chill, or a sense of coldness over the body particularly down the back, which lasted one or two hours and was followed by a preternatural heat over the whole body; the face flushed, and the eyes suffused—the pulse at the same time hurried and quick—prostration of strength—pain in the head and back, with sometimes soreness in the muscles of the legs and arms. The stomach toward the latter part of the season was irritable, rejecting in many instances every thing that was swallowed—the bowels costive, and the tongue, after the exacerbation had continued a few hours, put on a whitish or brown coat—the appetite for cold drinks came on sometimes with the chill, but more frequently after the reaction—the exacerbations continued from ten to twelve hours, when a gentle diaphoresis might be observed on the surface. If proper medical aid was interposed early, this form of the disease yielded kindly, but if neglected a few days, it would assume the characteristic symptoms of the inflammatory form. All the preceding symptoms, in an aggravated degree, were attendant on the inflammatory bilious fever. The tongue covered with a dark or brown colored coat, the eyes suffused and of a yellow cast, languid and dull; laborious respiration; delirium; corded quick pulse and sometimes very irregular; loss of appetite, nausea and vomiting; the stomach frequently throwing up a thick ropy fluid of a yellow or green color; the skin exceedingly hot and dry, and often of a yellow tinge; bowels costive and when evacuations were procured, large quantities of dark, offensive matter would come away; urine scanty and very high colored, resembling the ley of wood ashes. To the high excitement of this fever, local affections were frequently added. The brain, lungs, liver, spleen or bowels, was generally the seat of inflammation, attended with acute pains, and other well marked symptoms of inflammation in those parts. The brain was the part most frequently implicated, and from its relations and functions the most important. In addition to the symptoms just enumerated, we may add (in cases where the head was conspicuously affected) a deep and severe pulsating pain in the head, throbbing of the carotid and temporal arteries, an injected conjunctiva and pain in the bottom of the orbits, with great sensibility and intolerance of light. Obstinate watchfulness, intellectual confusion, delirium and coma now ensued, and as the disease advanced in an unobstructed course, the brain became oppressed and the exercise of all the functions suspended; the tongue became dry and black; a striking hebetude is discovered in all the senses; the eyes are now insensible or nearly so, with dilated pupils; the voice falters, the hands tremble; the patient moans; involuntary discharges of dark fœtid matter occur from the bowels; stertoreous breathing and convulsions close the scene.

Very few cases of this form of bilious fever proved fatal, when medical aid was resorted to in time. On the success of remediate agents, the stomach would become settled and composed, the remissions of longer continuance, the pulse soft and less frequent, the skin soft and moist; the tongue moist and clean around the edges, the inflammatory pains and sensorial disturbance would disappear, and the countenance comparatively a smile of composure, would presage a speedy convalescence. The liver was the next organ most generally implicated, and in all cases connected with yellowness of the eyes and skin, inflammation of that organ evidently predominated; the patient was distressed with a fullness and pain in the right hypochondriac region and under the shoulder blade in the course of the phrenic nerve, with clay or light colored stools. The other abdominal viscera before mentioned, were all (among the multiplicity of the afflicted) more or less seriously inflamed. This was indicated by pains in the regions which the respective organs occupy. In the two forms of bilious fever which I have endeavored to describe, as they appeared in the Wabash valley in the sickly year of 1834, it will be remembered that there was a high grade of action, or in other words, they were invariably attended with increased action of the heart and arteries, while the congestive form, which prevailed after the cool mornings and evenings of September, presented a striking difference in degree of arterial action. It was ushered in, as the other forms, with a cold fit of longer continuence; a partial reaction would succeed after a time, indicated by an increased temperature of the head and trunk, whilst the extremities remained cold: skin soft and clammy; pulse low, small and very compressible—during the exacerbation it would be full and soft; the temperature considerably below the natural standard over the whole system, or otherwise the heat was partial and confined to the head and trunk; the tongue after a few days put on a dark thick coat, especially in those cases where there was some increase of vascular action in the early stage: articulation indistinct and the countenance haggard; the stomach was sometimes irritable, the epigastric region sore, and extremely tender on pressure and inflated—bowels torpid and discharging dark and offensive matter, and in some instances involuntary discharges with delirium and stupor took place soon after the appearance of an attack. There was anxious respiration and low moaning, and the patient, when accosted, would complain of general soreness of the whole body, great prostration of strength; the eyes were dull and sunken and often glassy; and in all such cases as terminated unfavorably, involuntary stools with slight hemorrhages from the nostrils, fauces, gums, or bowels, announced the approach of death. I would here observe that the foregoing symptoms are those of the fevers which occurred in 1834, and are intended to apply to the well defined cases of the different forms here laid down, which are descriptive of our fevers in general up to the present period, with some modification in degree of violence, corresponding to the season.

*Treatment.*—As the treatment of the simple form so nearly resembles that of the inflammatory, I shall speak of them both under one head; and in the order in which they seem to be required, as nearly as the circumstances of the disease will permit, for indeed the remedies of the aggravated cases of the one and the slighter cases of the other, were the same, modified according to circumstances.

In a large majority of cases the lancet was strongly indicated and freely used early in the disease; blood was taken without regard to quantity and from a large orifice, until the pulse was reduced in force and frequency, and the pain in the head and difficult respiration relieved. Employed in this manner, the lancet will in many cases effect a speedy resolution of the paroxysm, and if it does not entirely prevent its recurance, it will certainly lesson its violence, reduce the temperature, alleviate the pain, and produce an increased susceptibility to the impressions of medicine. It should be repeated as often, and to as great an extent as the symptoms may require; those who were bled in the beginning of the fever, bore a repetition of the operation, at an advanced period, much better than when the previous evacuations had been omitted.

Emetics were next premised, and twenty grains of ipecacuanha with one or two of tartrate of antimony was generally prefered, and given so as to produce full vomiting, assisted in its operation by copious draughts of warm water. It is of much importance in the administration of this remedy, that it be given early in the commencement of the chill, when the case will admit of it, or immediately after the bleeding, while the system feels the impression occasioned by the loss of blood, before the disease has had time to reorganise its broken forces. By these means the diseased action was very often broken up, and convalescence speedily established. But it more frequently happened, that the disease pursued its course, and the next or succeeding paroxysm, was attended with extreme nausea and vomiting, when much relief was obtained from the effervescing mixture, mint tea, &c., with mustard sinapisms to the epigastric region and extremities.

As soon as the stomach would permit it, a cathartic of calomel and rhubarb, or calomel rhubarb and aloes, was given in a full dose, and repeated in such manner as to keep up continued evacuations of highly offensive and irritating matter from the bowels. I found the above combination to unload the bowels efficiently and promptly, producing in due course bilious evacuations which was beneficial in relieving the fever, pain, oppression, thirst and restlessness, when the general secretions succeeded that of the liver, and the disease soon yielded. In the advanced stage I found small doses of calomel, given at intervals of four or five hours, and determined to the bowels by infusion of senna and salts or Seidlitz powders, useful, supporting the biliary secretion and preserving the bowels in a soluble condition. Cathartics had a powerful effect in removing the congestive and inflammatory states of the viscera, as well as in subduing fever generally.

On the first exacerbation of fever, antimonials, or calomel in combination with ipecacuanha, was given every two, three, or four hours, according to circumstances, during the stage of excitement; constant nausea was maintained, until the violent symptoms moderated, when the medicines just mentioned were administered more sparingly. This course was pursued after the offensive contents of the bowls had been thoroughly discharged; it seldom failed to relieve the patient, and produce a general relaxation of the surface, with a soft moist skin. Blisters to the extremities, when much heat and dryness prevailed, proved very beneficial.

Diuretics were found of great importance in arresting the progress of disease. A close observer will invariably find that, soon after the urinary secretion is established, the liver and skin will both generally begin to yield their respective secretions, and that the disease will generally be unable to resist the efforts of these organs. To fill this indication nitrate of potass, spirits of nitre and digitalis were all occasionally used with good effect.

Epispastics were used for the double purpose of moderating febrile action, and to relieve local affections. In the one case they were applied to the wrists and ankles and down the spine, and in the other over the seat of pain.

*Tonics.*—As soon as the inflammatory stage had subsided, and a decided remission occurred, sulphate of quinine and other tonics were prescribed, with the happiest effect, until the restoration of health.

In the treatment of the congestive form of bilious fever, that prevailed in 1834, I was governed by two leading indications: 1st, the removal of the bilious symptoms present, with a view of relieving the congestion: 2d, the production of a reaction in the system, with the view of relieveing the congestion. To accomplish the first indication or to relieve the urgent bilious symptoms, active mercurial purgatives were prescribed, followed by castor oil, and enemata, which effectually evacuated the vitiated contents of the bowels; when the pulse, though weak at first, became stronger, and the rostrated energies of the system were thereby improved.

It was then necessary to continue the mercurials until the evacuations assumed a more natural appearance, and the urgent symptoms disappeared. For this purpose calomel was given in small doses; and where the patient was much prostrated, the addition of camphor was thought necessary. This combination was given every three or four hours, followed occasionally by mild purgatives, until the cure was effected. The small doses of calomel were prefered, in order to have the remedy act more immediately on the hepatic system, and correct more efficiently the borbid secretion of bile. To answer the second indication or to bring about reaction of the system, and relieve the congestion, in addition to the above course, an attempt was made to induce as much as possible an inflammatory disposition in the system. The warm bath assisted by powerful rubefacients such as tincture of capsicum sinapisms to the trunk and extremities, and where local congestion of any of the viscera existed along with the general disease, stimulating embrocations with friction were freely used, and lastly a sinapism applied over the part. This course seemed to obviate the necessity for internal stimulents, employed by some practitioners in congestive states of the system. They excited a warmth over the whole surface; the pulse became fuller, and more regular in its beats; and, aided by the repeated operation of purgatives, the delirium was removed, and the strength gradually regained. With regard to venesection, I would observe that I generally saw this form of fever in my country practice in the advanced stage, and so prostrated were the energies of the system, that it was entirely inadmissible. The remedy in particular oppressed states of the system may answer, when used with caution and discrimination; yet in the practice of 1834, I well recollect that the number of deaths was so large in proportion to the number bled, that it became proverbial among the people, that bleeding was death.

About the season of the year when congestive fever prevails, we have here a disease, itself congestive, commonly denominated *milk-sickness* or *trembles*, which in the fall of 1834

was, in this section, more extensive in its range, and more violent in its character. Whatever may be the cause of this distressing disease, I have been forcibly struck with its resemblance to many of our common autumnal diseases; and the more I see of it, the more firmly I incline to the opinion that it has, with them, a common cause, or is of miasmatic or atmospheric origin. The identity of this disease with the disorder in cattle called trembles I think probable, having witnessed horses, cattle, and dogs die with it, while attending on their owners with milk-sickness (as it is termed), though this does not prove, as is generally asserted, that it is produced by eating the milk, butter, or meat of cattle, apparently healthy, and which run at large in the woods. Are not animals subiect to many of the maladies incident to the human family? Individuals have been afflicted with it, that are known not to have used either butter, milk, or beef. A highly respectable Medical friend of mine, wbo has had much experience with this disease, was some time since attending upon a family with milk-sickness, who had no cattle, and had not made use of any of the above articles, while a family living near, whose cattle had this disease, and who had been using both milk and butter freely, not being aware for some time of the presence of disease among their stock, remained in perfect health. It is said that cattle kept in cultivated pastures escape the disease; this I believe to be generally true, so far as the subject has come under my observation in Indiana. Those farmers who have good cultivated pastures, are in general able to take good care of their stock, and feed well; and such cattle, with few if any exceptions, will escape it running in the woods; while on the contrary, I believe it will be found that poor, half-starved cattle will take it whether in pasture or out of it, if exposed to the exciting cause. I know a few families who have been living for many years in the very centre of the infested district, who are particularly careful of themselves and attentive to their cattle, and who have never suffered in a single instance, either in their families or in their stock, though the latter have run at large the whole time. That cattle in good condition are occasionally afflicted with this disease, is true; so are individuals in very comfortable circumstances occasionally afflicted with our fall fevers, but this does not prove that they are so liable as the poorer inhabitants of cold and uncomfortable dwellings. Thus, as I believe, it is not the confinement of the animal in good pasture or from the woods, that preserves its health, but the condition or nervous energy of the animal that resists the predominating cause.

This disease is ushered in by general prostration, particular after exercise, and a sensation of burning about the pyloric orifice of the stomach. After the lapse of two or three days, the debility and prostration increase, the burning sensation extends up the course of the esophagus, with nausea and incessant vomiting—there is obstinate constipation—the tongue is slightly coated but more generally preternaturally red; thirst intense, constantly craving cold water; pulse small and quick and sometimes hard and corded; with icy coldness of the extremities, and a countenance indicative of extreme suffering. The debility and prostration gradually increase to an alarming extent, most of the internal organs become involved, the secretions in general and particularly that of the liver are locked up, and all the morbid phenomena of the system are in full exercise. Towards the close of the disease, the pulse, that has been all along sinking, is now imperceptible, and there is hiccough, fainting, extreme coldness of the extremities, with delirium, coma, cold clammy sweats, frequent and involuntary discharges of black offensive stools, precisely similar to those evacuated in the last stage of typhoid fevers.

*Prognosis.*—A gradual cessation of the pain and burning sensation at the stomach, and of vomiting, with copious alvine discharges, a moist skin, with a return of the natural expression of the countenance, indicate a favorable termination of the disease. But, on the contrary, when the pain and burning of the stomach increase, when the vomiting continues almost incessantly, the bowels remaining confined, the pulse becoming nearly imperceptible, and the countenance cadaverous, the worst consequences are to be apprehended.

*Treatment.*—About three months since, I was called to a Mr. H., who was laboring under the disease for the ninth time in the last twelve years; he had had the disease for three or four days, and was in the second stage. When roused up, he would answer to any question promptly, but would instantly relapse into a kind of lethargy. He had all the usual symptoms before described. By means of two or three doses of calomel, rhubarb and aloes, with purgative enemata, and blisters to the epigastric region and extremities, he gradually recovered. After the lapse of a few weeks, he went to work and induced fatigue, when all the symptoms in full force again appeared. I used calomel in combination with extract of butternut, ol. ricini and turpentine, soda powders and purgative enemata, when he again recovered.

About the same time, I saw two young women who had had the disease every year for several years in succession, with symptoms as above. I used calomel and extract of butternut, purgative enemata, blisters to the epigastric region, and extremities, &c., under which treatment they both recovered. One of them went to work, and thereby induced fatigue, returned home in the evening, complained very little, threw herself on the bed, and died, before her relatives were aware of her danger. From the above, it will be evident that the first stage of the disease requires very little treatment. The only thing necessary is to enjoin perfect rest, and administer some of the milder laxatives, such as super tartrate potass and jalap, ol. ricini or sulph. magnesia, so as to keep up a loose state of the bowels.

The second stage requires a much more energetic practice, and from the character of the symptoms we would be led at once to the use of depletory remedies. Venesection in the commencement of this stage, is naturally suggested, and I have employed it, in very many cases with the happiest effect, though the all important remedy in the treatment of this disease, evidently consists, as much experience has taught me, in purging in such manner as to bring away copious evacuations, which generally allays the gastric irritability, and restores the lost balance of the circulation. The greatest difficulty in obtaining this object, is occasioned by the constant vomiting which is generally relieved after the action of a blister applied over the region of the stomach.

*New Harmony*, 1843.

